# COVID-19 and Acute Kidney Injury: A Systematic Review and Meta-Analysis

**DOI:** 10.3390/pathogens9121052

**Published:** 2020-12-15

**Authors:** Fabrizio Fabrizi, Carlo M. Alfieri, Roberta Cerutti, Giovanna Lunghi, Piergiorgio Messa

**Affiliations:** 1 Division of Nephrology, Fondazione IRCCS Ca’ Granda Ospedale Maggiore Policlinico, 20122 Milano, Italy; alfieri@policlinico.mi.it (C.M.A.); cerutti@policlinico.mi.it (R.C.); messa@policlinico.mi.it (P.M.); 2 Department of Clinical Sciences and Community Health, University of Milan, 20133 Milan, Italy; 3 Virology Unit, Fondazione IRCCS Ca’ Granda Ospedale Maggiore Policlinico, 20122 Milano, Italy; lunghi@policlinico.mi.it

**Keywords:** acute kidney injury, renal replacement therapy, COVID-19, mortality, SARS-CoV-2, severe disease

## Abstract

Background: coronavirus disease 2019 (COVID-19), caused by severe acute respiratory syndrome—coronavirus-2 (SARS-CoV-2)—is an ongoing pandemic with high morbidity and mortality rates. Preliminary evidence suggests that acute kidney injury (AKI) is uncommon in patients with COVID-19 and associated with poor outcomes. Study aims and design: we performed a systematic review of the literature with a meta-analysis of clinical studies to evaluate the frequency of AKI and dialysis requirement in patients who underwent hospitalization due to COVID-19. The incidence of AKI according to the death risk was calculated in these patients. The random-effects model of DerSimonian and Laird was adopted, with heterogeneity and stratified analyses. Results: thirty-nine clinical studies (n = 25,566 unique patients) were retrieved. The pooled incidence of AKI was 0.154 (95% CI, 0.107; 0.201; *p* < 0.0001) across the studies. Significant heterogeneity was found (*p* = 0.0001). The overall frequency of COVID-19-positive patients who underwent renal replacement therapy (RRT) was 0.043 (95% CI, 0.031; 0.055; *p* < 0.0001); no publication bias was found (Egger’s test, *p* = 0.11). The pooled estimate of AKI incidence in patients with severe COVID-19 was 0.53 (95% CI, 0.427; 0.633) and heterogeneity occurred (Q = 621.08, I2 = 97.26, *p* = 0.0001). According to our meta-regression, age (*p* < 0.007) and arterial hypertension (*p* < 0.001) were associated with AKI occurrence in hospitalized COVID-19 positive patients. The odds ratio (OR) for the incidence of AKI in deceased COVID-19 positive patients was greater than among survivors, 15.4 (95% CI, 20.99; 11.4; *p* < 0.001). Conclusions: AKI is a common complication in hospitalized COVID-19 positive patients. Additional studies are under way to assess the risk of AKI in COVID-19 patients and to deepen the mechanisms of kidney injury.

## 1. Introduction

The newly discovered Severe Acute Respiratory Syndrome Coronavirus 2 (SARS-CoV-2, previously named as 2019 novel coronavirus or 2019-nCoV) has been identified as the pathogen of Coronavirus Disease 2019 (COVID-19). The novel virus identified in Wuhan, China, in December 2019 and has spread rapidly all over the world. The World Health Organization (WHO) declared COVID-19 a pandemic in March 2020 [1]. Typical patient clinical manifestations included fever, unproductive cough, dyspnea, fatigue, normal or lowered white blood cell count, and imaging evidence of pneumonia. The clinical course of infection by SARS-CoV-2 is widely unpredictable and variable, ranging from asymptomatic infection to multi-organ systemic failure and death. 

SARS-CoV-2 belongs to the large family of viruses named coronaviruses. Other coronaviruses are capable of causing illnesses including human severe acute respiratory syndrome coronavirus (SARS-CoV) and Middle East Respiratory Syndrome Coronavirus (MERS-CoV). It appears that SARS-CoV-2 has a lower fatality incidence but a higher infection rate than SARS-CoV and MERS-CoV which caused previous epidemics [2].

Several epidemiological studies have shown that patients with comorbidities, such as diabetes, arterial hypertension, metabolic syndrome, and cardiovascular disease as well as older individuals are more susceptible to exhibit symptoms with SARS-CoV-2 infection and carry an increased risk of progression to severe disease [3]. The death rate, in addition, is high when accompanied by organ dysfunction such as in the lungs or kidneys [3]. A close association between acute kidney injury (AKI) and coronavirus infection has been recorded in SARS-CoV and MERS-CoV epidemics. It has been shown that AKI developed in 5% to 15% cases and gave a high death rate (70% to 90%) in SARS and MERS-CoV infections [4,5]. 

There is limited information regarding the development of AKI in patients with COVID-19. We performed a systematic review with a meta-analysis of clinical studies to assess the incidence of AKI in hospitalized COVID-19 population. We evaluated the association of AKI with the outcomes of severe acute respiratory syndrome coronavirus 2 (SARS-CoV-2). 

## 2. Materials and Methods

### 2.1. Search Strategy and Data Extraction

The study was made according to the Preferred Reporting Items for Systematic Reviews and Meta-Analyses (PRISMA) recommendations and criteria for the reporting of meta-analysis guidelines [6]. National Library of Medicine MEDLINE and manual searches were combined, as it had been previously demonstrated that a MEDLINE search alone may not be sensitive enough [7]. The following key words were adopted: (‘COVID-19’ OR ‘Severe Acute Respiratory Syndrome Coronavirus 2’ OR ‘SARS-CoV-2’ OR ‘2019-nCoV’ OR ‘novel coronavirus’) AND (‘Acute Kidney Injury’ OR ‘Acute Renal Impairment’ OR ‘Acute Renal Failure’ OR ‘Renal Replacement Therapy’) AND (‘Mortality’ OR ‘Severe Disease’ OR ‘Death Rate’). General reviews, references from published clinical trials, letters to pharmacological companies, and current contents were also used. All articles were retrieved by a search from 1 December 2019 to 30 June 2020. Data extraction was performed independently by two investigators (F. F., and R. C.), and consensus was obtained for all data. Studies were compared to eliminate duplicate reports for the same patients, which included contact with investigators when necessary. Eligibility and exclusion criteria were pre-specified. 

### 2.2. Criteria for Inclusion

The included studies had to meet the following criteria: (I) clinical trials including cohort or case-control or descriptive studies; (II) human studies involving the identification of COVID-19 patients; (III) studies providing evidence on the clinical features of patients with COVID-19. Patients who were admitted to ICU and non-ICU were also categorized into a severe and non-severe subgroup. Studies which were preprint were also included.

### 2.3. Ineligible Studies 

Studies from which data extraction was not possible were excluded. Studies focusing on pregnant patients or other coronaviruses, such as MERS or SARS, were excluded. We excluded studies which did not give information on grouping between severe and non-severe patients if unable to obtain data from the investigators. Studies that were only published as abstracts, letters, case reports or interim reports were excluded; review articles were not evaluated for the current review. 

### 2.4. End-Points of Interest

Primary outcomes of interest were the pooled incidence of AKI and the requirement of renal replacement therapy (RRT), in hospitalized patients with COVID-19. An additional end-point was the AKI occurrence in hospitalized patients with severe COVID-19. The impact of AKI on the death risk of COVID-19 patients was addressed by calculating the summary estimate for unadjusted or adjusted death risk. As detailed below, the adjusted death risk was generated by multivariate analysis in a subset of reports. The adjusted relative risks (aRR) of all-cause mortality was calculated in each study. 

### 2.5. Statistical Methods

The summary estimate of the incidence of AKI and the need of RRT was calculated. We computed fixed and random effect estimates and the random-effects model of Der Simonian and Laird was adopted if moderate to severe heterogeneity occurred [8]. To assess the between-study heterogeneity, we used Cochran’s *Q* test (*p* > 0.10 for statistical significance) and *I*^2^ test [9]. To further explore the origin of heterogeneity, we restricted the analysis to subgroups of studies defined by study characteristics such as the country of origin (China, United States of America), and study design (retrospective or not), among others. We made a funnel plot to detect a publication bias in the relation exposure at hand; publication bias was calculated by Egger’s test. Meta-regression was carried out to assess the independent effect of continuous covariates on the incidence of AKI in hospitalized patients with COVID-19. We adopted the odds ratio (OR) with 95% CI for the dichotomous outcomes. In a subset of reports, a summary estimate of the adjusted RR of all-cause mortality among hospitalized COVID-19 patients who developed AKI compared with those who did not was generated by weighting the study-specific RR’s (by the inverse of the variance). The aRR was calculated by multivariate analysis (i.e., after adjustment for potential confounders such as comorbidities and complications). R*i* (the proportion of total variance due to between studies variance) was adopted to take into account the heterogeneity. All the statistical analyses were performed using Rev Man (Review Manager) 5.0, The Cochrane Collaboration (2020), Comprehensive meta-analysis (CMA 1.0), and HEpiMA, version 2.1.3 [10]. The 5% significance level was adopted for alpha risk. Every estimate was recorded with 95% confidence intervals (CIs).

## 3. Results

### 3.1. Literature Review

**Figure 1:**
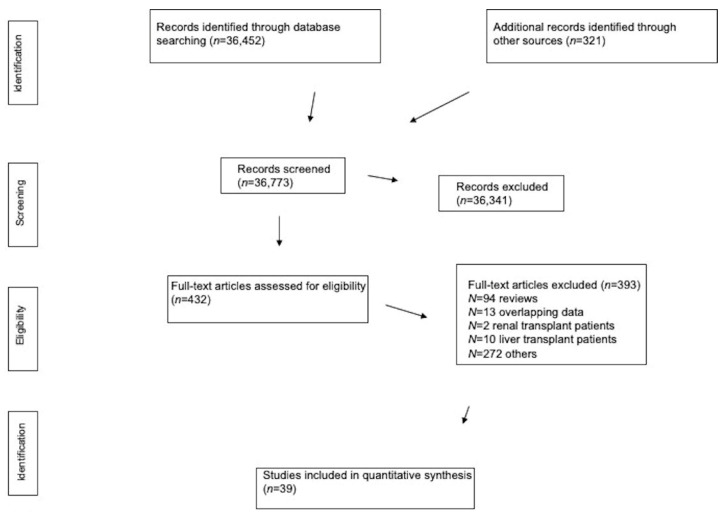
Flow diagram of the study selection.

Our electronic and manual searches identified 432 full-text articles that were considered potentially relevant and selected for full-text review. A complete list of the 432 full-text articles reviewed is available from the authors on request. We excluded 393 full-text articles, as detailed in Figure 1. 

We included a total of 39 reports giving information on 25,566 patients who had been admitted to tertiary hospitals all over the world and were diagnosed with COVID-19 (Figure 1). There was a 100% concordance between reviewers with respect to the final inclusion and exclusion of studies based on the predefined exclusion criteria.

### 3.2. Patient Characteristics

According to the design of the study, two sets of reports were identified. The first set was constituted by studies listed in Table 1 and Table 2 and Supplementary Table S1 (*n* = 22; *n* = 8792 patients) which evaluated the frequency of AKI based on the severity of COVID-19 [11,12,13,14,15,16,17,18,19,20,21,22,23,24,25,26,27,28,29,30,31,32]. The frequency of chronic obstructive pulmonary disease (COPD) ranged between 1.1 and 13%, and the chronic liver disease (CLD) rate between 0.9% and 11.8%. 

The second set included reports shown in Table 3 and Table 4 and Supplementary Table S2 (*n* = 20, *n* = 16,774 patients) which assessed the impact of AKI development upon the outcomes (death rate) of COVID-19 patients [33,34,35,36,37,38,39,40,41,42,43,44,45,46,47,48,49]. Some (*n* = 3) studies gave information on both relationships [16,24,28]. The frequency of COPD varied between 1.9 and 12%, and the rate of CLD between 0.52% and 6.3%. 

Table 1 and Table 3 report the list of studies evaluated, the countries where they were carried out, the reference year and some demographic data. All studies were conducted between January and June 2020. As listed in Table 1, Table 2, Table 3 and Table 4, the majority of reports were from China (*n* = 30), and the others from the USA (*n* = 7) and South Korea (*n* = 2), respectively. The frequency of male patients ranged from 38.8% to 75%, and the mean age from 47 to 69 years. Comorbidities (arterial hypertension, diabetes mellitus, chronic kidney disease (CKD), cardiovascular disease) have been recorded in Table 2 and Table 4 and Supplementary Tables S1 and S2. The majority of the studies adopted the definition of AKI according to the 2012 Kidney Disease: Improving Global Outcomes (KDIGO) clinical practice guidelines, where AKI is defined as any of the following: increase in serum creatinine by ≥0.3 mg/dL (≥26.5 μmol/L) within 48 h; or increase in serum creatinine to ≥1.5 times baseline, which is known or presumed to have occurred within the prior 7 days; or < urine volume 0.5 mL/kg/h for 6 h [50]. In some papers, the definition of AKI was not mentioned [16,36,38,40,46]. 

### 3.3. AKI Incidence: Primary and Stratified Analysis

**Figure 2:**
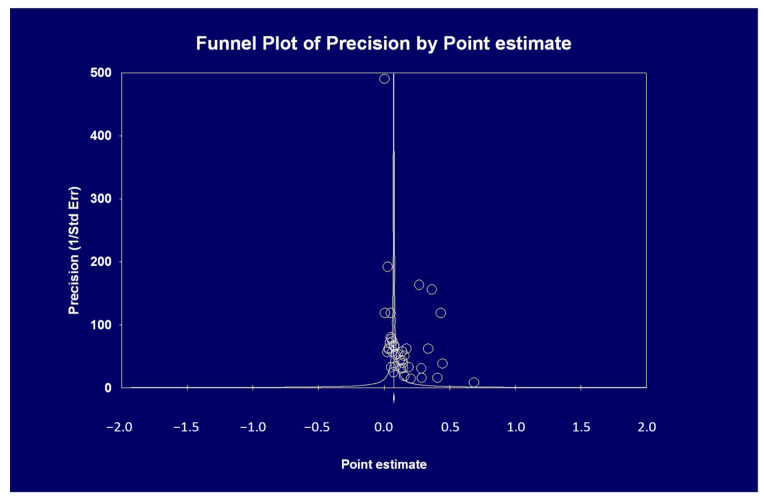
The pooled incidence of AKI in COVID-19 positive patients during their hospital stay was 0.154 (see text); the funnel plot asymmetry suggests the occurrence of publication bias (this is confirmed by Egger’s test, *p* = 0.025).

Table 5 shows that the summary estimate for the occurrence of AKI across the identified trials was 0.154 (95% CI, 0.107, 0.201). Significant heterogeneity was found (Table 5) (*p* = 0.0001). The Egger’s regression intercept shows that there was publication bias (*p* = 0.025) (Figure 2). 

Stratified analyses were undertaken to explain the heterogeneity across studies (Table 5). The analysis by the fixed-effects model yielded very similar findings to the random-effects model (data not shown). 

**Figure 3:**
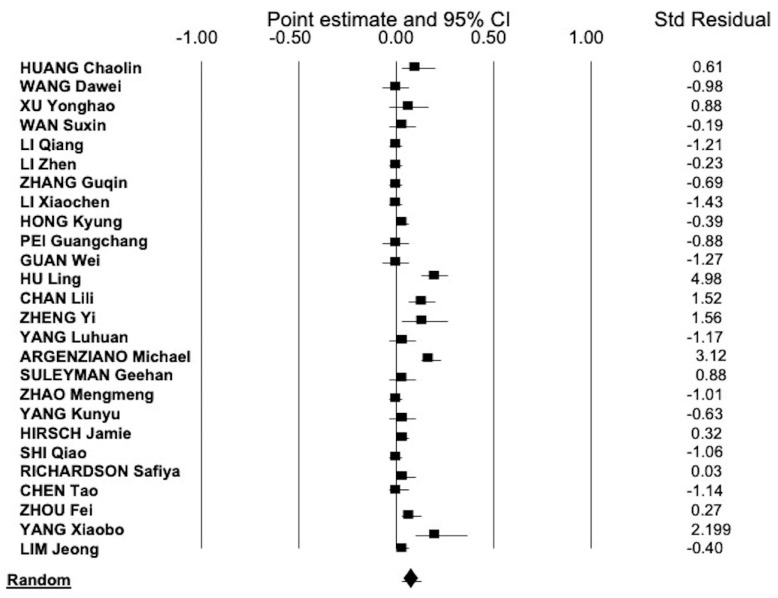
The summary estimate of the frequency of renal replacement therapy (RRT)-dependent AKI in hospitalized patients with COVID-19 was 0.043 (95% CI, 0.031; 0.055) (random-effects model).

The overall estimate for the frequency of COVID-19 positive patients who had AKI and underwent RRT during their hospital stay was 0.043 (95% CI, 0.031; 0.055) (Figure 3). Heterogeneity occurred, *Q* = 596,8 d(f) = 25, *I*^2^ = 95.8 (*p* = 0.0001). No publication bias was found (Egger’s test, *p* = 0.11). 

### 3.4. AKI Incidence and Severe Disease in COVID-19-Positive Patients

**Figure 4:**
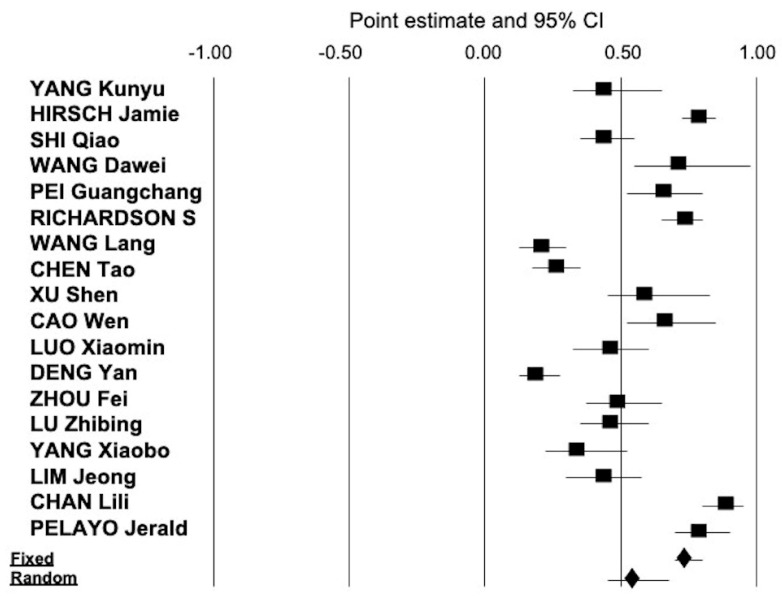
The summary estimate of the frequency of AKI in hospitalized patients with severe COVID-19 was 0.53 (95% CI, 0.427; 0.633) (random-effects model).

As reported in Figure 4, the pooled estimate of frequency of AKI in patients with severe COVID-19 was 0.53 (95% CI, 0.427; 0.633). There was consistent heterogeneity (*Q* = 621.08, *I*^2^ = 97.26, *p* = 0.0001). 

### 3.5. AKI Incidence and Death Rate in COVID-19 Positive Patients

**Figure 5:**
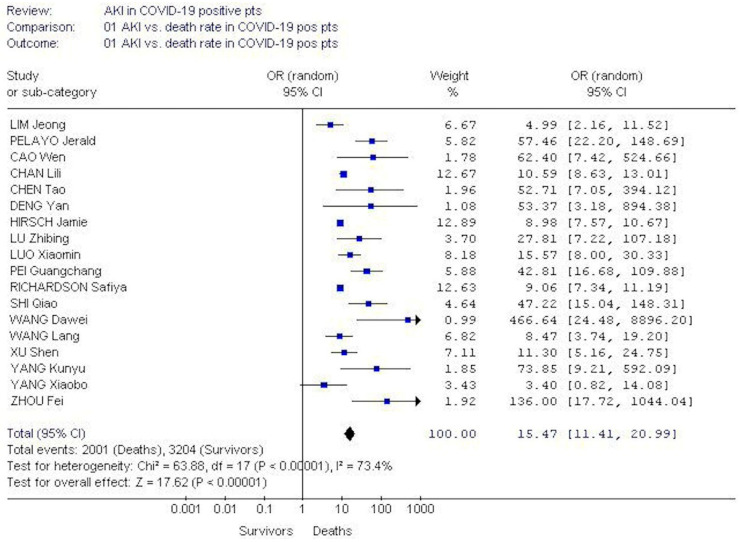
The pooled OR of AKI incidence was greater in deceased COVID-19-positive patients than among survivors during their hospital stay, 15.4 (95% CI, 20.99; 11.4) (random-effects model).

**Figure 6:**
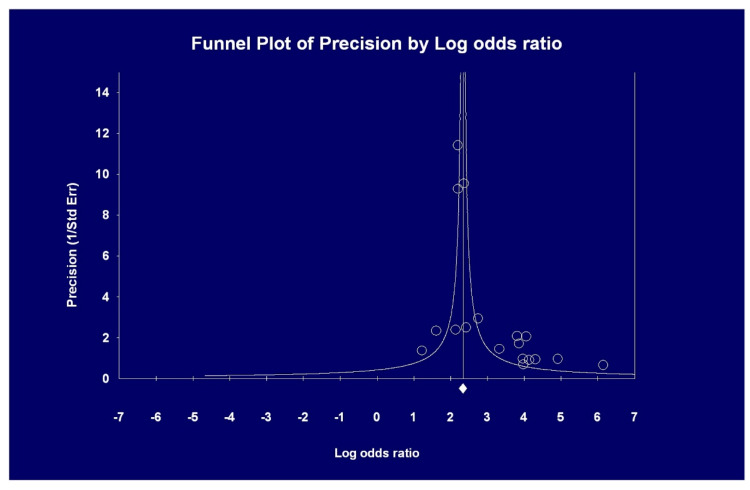
Pooled odds ratio (OR) of AKI in deceased vs. survivor patients with COVID 19 (see text); the asymmetry of the funnel plot indicates the possibility of publication bias and this is confirmed by the Egger ‘s test (*p* = 0.0016).

The pooled OR of AKI incidence among deceased COVID-19 positive patients was greater than among survivors, 15.4 (95% CI, 20.99; 11.4). Test for heterogeneity was significant (*p* = 0.00001) (Figure 5). Publication bias occurred (Egger’s regression, *p* = 0.0016) (Figure 6). 

**Figure 7:**
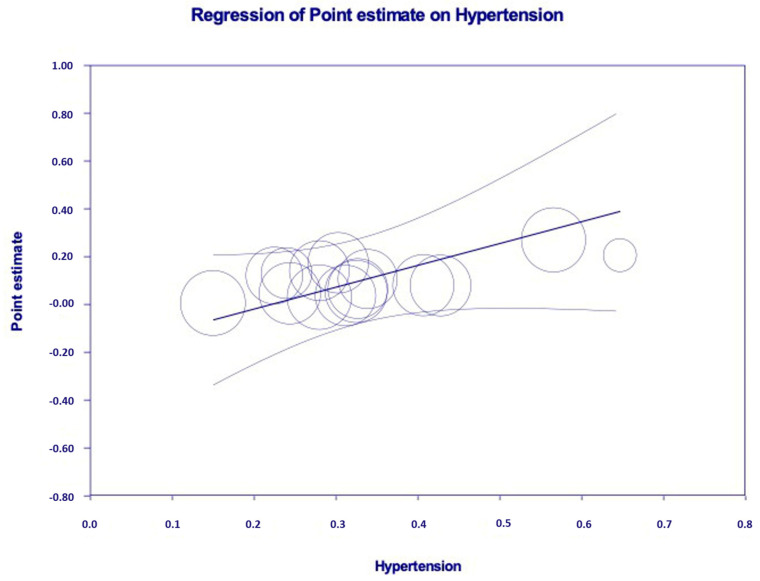
Independent and significant relationship between arterial hypertension and the frequency of AKI (*p* = 0.001) in COVID-19 positive patients who underwent hospitalization (meta-regression analysis).

Some authors (*n* = 5, *n* = 5435 unique patients) evaluated the association between AKI and death risk by multivariate analysis. As shown in Table 6, the link between AKI and death risk remained significant in many comparisons. The results of meta-regression are reported in Table 7. Age (*p* = 0.007) and arterial hypertension (*p* = 0.001) are significantly associated with the frequency of AKI. The independent and significant relationship between the frequency of AKI and arterial hypertension according to meta-regression is shown in Figure 7.

## 4. Discussion

Controversy exists about kidney involvement in COVID-19-positive patients. Preliminary evidence indicated that the frequency of kidney disease in the COVID-19 population was negligible and limited interest has been given to the incidence of AKI in patients with COVID-19 [3]. Additional studies have highlighted the frequency of kidney abnormalities in patients with COVID-19 [51]. The current systematic review of the scientific literature with a meta-analysis of clinical studies indicated that the incidence of AKI in patients with COVID-19 during their hospital stay was common (around 15%). The frequency of AKI among patients with severe COVID-19 was much greater (around 50%). We noted important heterogeneity that could be explained by numerous factors such as patient characteristics, severity of illness, differences in daily clinical practice (regarding fluid management, ventilation options and medications, among others). 

According to our meta-regression analysis, some comorbidities (age and arterial hypertension) were significantly related to AKI occurrence and this is in keeping with the evidence on the development of AKI in patients without COVID-19. 

The pathophysiological mechanisms which are responsible for COVID-19-related AKI are yet to be discovered [51]. Unspecific mechanisms exist including comorbidities (diabetes mellitus, arterial hypertension, and others) which confer vulnerability to kidneys, nephrotoxic drugs or contrast media, hypovolemic conditions and subsequent pre-renal AKI. Multiorgan involvement is common in patients with COVID-19 including damage to kidneys, heart, and gastrointestinal tract; this mirrors the presence of the ACE2 receptors in various organs which serve as an entrance door for SARS-CoV-2. It has been hypothesized that the development of AKI in COVID-19 patients include viral cytopathic activity, hypoperfusion, cytokine storm, and microvascular thrombosis [51]. Alternatively, patients with severe SARS-CoV-2 infection frequently show acute respiratory distress syndrome (ARDS); severe hypoxemia or high intra-thoracic pressures have been linked to AKI in the ARDS population [52]. COVID-19-specific mechanisms include the entry of SARS-CoV-2 into the kidneys and the binding of SARS-CoV-2 with the ACE2 receptor on the cell membrane of the host cells; in the kidneys, the ACE2 receptor is expressed in the apical brush borders of the proximal tubules as well as podocytes [53]. In addition, COVID-19 promotes an imbalanced activation of the renin–angiotensin–aldosterone system (RAAS), which induces the downregulation of the membrane-bound ACE2 receptor that promotes the accumulation of angiotensin II by lowering its degradation into angiotensin 1–7. Imbalanced RAAS activation leads to inflammation, vasoconstriction and fibrosis at the kidney level [54]. Some studies have suggested that ACE inhibitors and angiotensin receptor blockers (ARBs) may improve ACE2 expression and therefore increase the susceptibility of patients to SARS-CoV-2 infection [55]. High levels of inflammatory cytokines have been noted in severe COVID-19 patients and may participate to AKI in these patients [35]. 

The current meta-analysis is flawed by numerous issues. Most studies included in this study had retrospective design; there were no RCTs. It is well known that prospective studies having data at baseline and over follow-up provide better evidence. Second, individual data from each study (e.g., ‘patient-level data’) were not available; thus, it was impossible to make our own adjustments. An additional limitation is given by the occurrence of publication bias: negative studies are less likely to be published. In addition, an enormous body of data is rapidly accumulating on COVID-19 patients, including those with kidney disease, and this clearly makes difficult the retrieval of the whole evidence on the subject. We have not adopted the studies published as abstracts or letters as information presented in this format as these are not of high quality. 

In conclusion, this meta-analysis of clinical studies shows that AKI is common in COVID-19-positive patients during their hospital stay. The frequency of AKI was much greater in patients with severe disease. There is a consistent relationship between the development of AKI and unsatisfactory outcomes (death rate) in hospitalized patients with COVID-19. 

## Figures and Tables

**Table 1:** Acute kidney injury (AKI) according to severe COVID-19: observational studies.

Authors (Ref.)	Country	Publication Date	Patients, *n*
HUANG Chaolin et al. [11]	Jin Yin-tan Hospital (Wuhan, China)	24 January 2020	41
WANG Dawei et al. [12]	Zhongnan Hospital (Wuhan, China)	7 February 2020	138
XU Yonghao et al. [13]	Seven Hospitals (Guangdong, China)	6 March 2020	45
WAN Suxin et al. [14]	Three Gorges Central Hospital (Chongqing, China)	21 March 2020	135
LI Qiang et al. [15]	Shanghai Hospital (Shangai, China)	24 March 2020	325
XU Shen et al. [16]	Two Hospitals (Hubei and Anhui, China)	26 March 2020	355
LI Zhen et al. [17]	Four Hospitals (Wuhan, Huangshi, Chongqing, China)	27 March 2020	193
ZHAO Wen et al. [18]	YouAn Hospital (Beijing, China)	30 March 2020	77
WANG Luwen et al. [19]	Remnin Hospital (Wuhan, China)	31 March 2020	116
ZHANG Guqin et al. [20]	Zhongnan Hospital (Wuhan, China)	9 April 2020	221
LI Xiaochen et al. [21]	Tongji Hospital (Wuhan, China)	12 April 2020	548
JIANG Xiufeng et al. [22]	Wuxi Fifth People’s Hospital (Jiangsu, China)	14 April 2020	55
HONG Kyung Soo et al. [23]	Yeungnam Hospital (Daegu, Korea)	24 April 2020	98
PEI Guangchang et al. [24]	Tongji Hospital (Wuhan, China)	28 April 2020	333
AGGARWAL Saraubah et al. [25]	UnityPoint Clinic (Des Moines, USA)	29 April 2020	16
GUAN Wej et al. [26]	552 hospitals (Mainland, China)	30 April 2020	1099
HU Ling et al. [27]	Tianyou Hospital (Wuhan, China)	3 May 2020	323
CHAN Lili et al. [28]	5 hospitals (New York City, USA)	8 May 2020	3235
ZHENG Yi et al. [29]	First Hospital (Hangzhou, China)	20 May 2020	34
YANG Luhuan et al. [30]	Yichang Hospital, China	26 May 2020	200
ARGENZIANO Michael et al. [31]	NYP/CUIMC (New York Presbyterian/Columbia University Irving Medical Center) (New York City, USA)	29 May 2020	850
SULEYMAN Geehan et al. [32]	HFHS (Henry Ford Health System) (Detroit, USA)	16 June 2020	355

**Table 2:** AKI according to severe COVID-19: patient characteristics.

Authors	Males, *n*	Age, Years	Chronic Kidney Disease (CKD)
HUANG Chaolin et al.	30 (73%)	49 (41–58)	NA
WANG Dawei et al.	75 (54.3%)	56 (42–68)	4 (2.9%)
XU Yonghao et al.	29 (64.4%)	56.7 ± 15.4	NA
WAN Suxin et al.	72 (53.3%)	47 (36–55)	NA
LI Qiang et al.	167 (51.4%)	51 (36–64)	4 (1.2%)
XU Shen et al.	193 (54.3%)	NA	NA
LI Zhen et al.	95 (49%)	57 (46–67)	NA
ZHAO Wen et al.	34 (44.2%)	52 ± 20	5 (6.5%)
WANG Luwen et al.	67 (57.8%)	54 (38–69)	5 (4.3%
ZHANG Guqin et al.	108 (48.9%)	55 (39–66)	6 (2.7%)
LI Xiaochen et al.	279 (50.9%)	60 (48–69)	10 (1.8%)
JIANG Xiufeng et al.	27 (49.1%)	45 (27–60)	1 (1.8%)
HONG Kyung et al.	38 (38.8%)	55.4 ± 17.1	NA
PEI Guangchang et al.	182 (54.7%)	56.3 ± 13.4	NA
GUAN Wej et al.	637 (58.1%)	47 (35–58)	8 (0.8%)
AGGARWAL Saraubah et al.	12 (75%)	67 (38–95)	6 (37.5%)
HU Ling et al.	166 (51.4%)	61 (23–91)	7 (2.2%)
CHAN Lili et al.	1868 (57.7%)	66.5 (55–78)	323 (10%)
ZHENG Yi et al.	23 (67.6%)	66 (58–76)	2 (5.9%)
YANG Luhuan et al.	98 (49%)	55 ± 17.1	3 (1.5%)
ARGENZIANO Michael et al.	511 (60%)	63 (50–75)	NA
SULEYMAN Geehan et al.	165 (46.5%)	61.8 ± 15.3	161 (45.3%)

**Table 3:** AKI and death risk in COVID-19: observational studies.

Authors	Country	Publication Date	Patients, *n*
YANG Xiaobo et al. [33]	Jin Yin-tan Hospital (Wuhan, China)	21 February 2020	52
LU Zhibing et al. [34]	Zhongnan and Seventh Hospitals (Wuhan, China)	3 March 2020	123
ZHOU Fei et al. [35]	Jin Yin-tan and Pulmonary Hospitals (Wuhan, China)	9 March 2020	191
DENG Yan et al. [36]	Tongji Hospital (Wuhan, China)	20 March 2020	225
CHENG Yichun et al. [37]	Tongji Hospital, (Wuhan, China)	20 March 2020	701
LUO Xiaomin et al. [38]	Renmin Hospital (Wuhan, China)	23 March 2020	403
CAO Wen et al. [39]	Jinyintan Hospital (Wuhan, China)	24 March 2020	61
XU Shen et al. [16]	Huazhong (Hubei) and Fuyang (Anhui) Hospitals (China)	26 March 2020	355
CHEN Tao et al. [40]	Tongji Hospital (Wuhan, China)	26 March 2020	274
WANG Lang et al. [41]	Renmin Hospital (Wuhan, China)	30 March 2020	339
RICHARDSON Safiya et al. [42]	New York City (USA)	22 April 2020	5700
PEI Guangchang et al. [24]	Tongji Hospital (Wuhan, China)	28 April 2020	333
CHAN Lili et al. [28]	NYC, USA	8 May 2020	3235
WANG Dawei and Yin D. et al. [43]	Zhongnan and Xishui Hospitals (Wuhan, China)	30 April 2020	107
SHI Qiao et al. [44]	Renmin and Zhongnan Hospitals (Wuhan, China)	14 May 2020	1561
HIRSCH Jamie et al. [45]	NYC, (USA)	16 May 2020	5449
YANG Kunyu et al. [46]	Local Hospitals (*n*=9) (Hubei, China)	29 May 2020	205
LIM, Jeong-Hoon et al. [47]	Kyungpook University Hospital (Daegu, Korea)	3 June 2020	160
ZHAO Mengmeng et al. [48]	Remnin Hospital (Wuhan, China)	4 June 2020	1000
PELAYO J. et al. [49]	Einstein Medical College (Philadelphia, USA)	18 June 2020	223

**Table 4:** AKI and death risk in COVID-19: patient characteristics.

Authors	Male, *n*	Age, Years	CKD
YANG Xiaobo et al.	35 (67%)	59.7 ± 13.3	NA
LU Zhibing et al.	61 (49.5%)	57.8 ± 12.7	7 (5.7%)
ZHOU Fei et al.	119 (62%)	56 (46–67)	2 (1.0%)
DENG Yan et al.	124 (55.1%)	54 (33–74)	NA
CHENG Yichun et al.	367 (52.4%)	63 (50–71)	14 (2.0%)
LUO Xiaomin et al.	193 (47.9%)	56 (39–68)	7 (1.7%)
CAO Wen et al.	36 (59%)	61 (48–70)	NA
XU Shen et al.	NA	NA	NA
CHEN Tao et al.	171 (62.4%)	62 (44–70)	4 (1.4%)
WANG Lang et al.	166 (48.9%)	69 (65–76)	13 (3.8%)
RICHARDSON Safiya et al.	3437 (60.3%)	63 (52–75)	268 (4.7%)
PEI Guangchang et al.	182 (54.7%)	56.3 ± 13.4	NA
CHAN Lili et al.	1868 (52.8%)	66.5 (55–78)	323 (10%)
WANG Dawei and Yin D. et al.	57 (53.3%)	51 (36–65)	3 (2.8%)
SHI Qiao et al.	150 (49%)	64.5 (56–72)	12 (3.9%)
HIRSCH Jamie et al.	3317 (60.9%)	64 (52–75)	NA
YANG Kunyu et al.	96 (47%)	63 (56–70)	4 (1.9%)
LIM, Jeong-Hoon et al.	86 (53%)	68.5 (24–98)	NA
ZHAO Mengmeng et al.	466 (46.6%)	61 (46, 70)	24 (2.4%)
PELAYO Jerald et al.	108 (48.4%)	65.9	39 (17.8%)

**Table 5:** Summary estimates for unadjusted point estimates of AKI in hospitalized COVID-19 patients.

	Study, *n*	Point Estimate (Random Effects Model) (95% CI)	*p*-Value (By *Q* Test)	*I* ^2^
All studies	39	0.154 (0.107; 0.201)	0.0001 (6929,1)	99.4%
Studies (China)	30	0.094 (0.075; 0.114)	0.0001 (567.09)	94.8%
US studies	7	0.353 (0.286; 0.42)	0.0001 (374.9)	98.4%
Studies (Korea)	2	0.139 (0.044; 0.233)	0.023 (5.168)	80.6%
Retrospective studies	28	0.17 (0.109; 0.23)	0.0001 (2433.1)	98.8%
Population-based studies	4	0.268 (0.04; 0.496)	0.0001 (6049.3)	99.9%
Small studies *	10	0.17 (0.098; 0.243)	0.0001 (108.4)	92.6%

* small studies (studies with size < 100 pts).

**Table 6:** Summary estimates for adjusted relative risks (aRR, adjusted relative risk by Cox proportional hazard model) of all-cause mortality among hospitalized patients with COVID-19.

Authors	Study, *n*	Fixed Effects aRR (95% CI)	Random Effects aRR (95% CI)	R*i*	*p*-Value (by *Q*-Test)
All studies	5	5.24 (4.31; 6.38)	2.80 (0.96, 8.13)	0.97	0.00001
Studies (China)	3	1.62 (1.13; 2.32)	1.66 (0.56; 4.91)	0.89	0.00001
Studies (others from China)	2	8.67 (6.86; 10.96)	6.27 (2.43; 16.19)	0.93	0.0127

Chan L. et al. [28]: aRR adjusted for age, gender, comorbidities including hypertension, congestive heart failure, diabetes mellitus, liver disease, peripheral vascular disease, chronic kidney disease, laboratory values including white blood cell count, lymphocyte percentage, hemoglobin, platelets, sodium, potassium, chloride, bicarbonate, urea, creatinine, aspartate aminostransferase, alanine aminotransferase, alkaline phosphatase, albumin, and vitals (including systolic blood pressure, diastolic BP, heart rate, respiratory rate, oxygen saturation). Cheng Y. et al. [37]: aRR adjusted for age, gender, disease severity, any comorbidity (CKD, chronic obstructive pulmonary disease (COPD), hypertension, diabetes, and tumor), and lymphocyte count. Wang L. et al. [41]: aRR adjusted for age, acute cardiac injury, arrhythmia, acute respiratory distress syndrome (ARDS), cardiac insufficiency, bacterial infection). Lim J. et al. [47]: aRR adjusted for age, gender, hypertension, diabetes. Zhao M. et al. [48]: aRR adjusted for age, shock, acute cardiac injury, acute liver injury, and number of complications (1, 2 or more).

**Table 7:** Meta-regression: impact of continuous covariates on the outcome of interest (AKI rate among hospitalized patients with COVID-19).

Covariate	Coefficient	Standard Error	95% CI	*Z*-Value	*p*-Value
Intercept	0.616	0.330	−0.031; 1.265	1.86	0.062
Diabetes mellitus	−0.179	0.185	−0.5426; 0.184	−0.97	0.333
Male	−0.110	0.330	−0.758; 0.5379	−0.33	0.739
Age	−0.012	0.004	−0.021; −0.003	−2.7	0.007
Hypertension	0.913	0.286	0.352; 1.475	3.19	0.001
cardiovascular disease	0.160	0.099	−0.034; 0.356	1.61	0.107
CKD	−1.352	1.041	−3.393; 0.687	−1.3	0.193
COPD	1.297	0.908	−0.482; 3.076	1.43	0.153
chronic liver disease	−0.523	0.607	−1.714; 0.666	−0.86	0.388

## Supplementary Materials

The following are available online at https://www.mdpi.com/2076-0817/9/12/1052/s1, Table S1: AKI according to severe COVID-19: Comorbidities, Table S2: AKI and death risk in COVID-19: Comorbidities.

## Abbreviations

ACE	Angiotensin converting enzyme
AKI	Acute kidney injury
ARB	Angiotensin receptor blocker
ARDS	Acute respiratory distress syndrome
CI	Confidence intervals
CLD	Chronic liver disease
COPD	Chronic obstructive pulmonary disease
COVID-19	Coronavirus disease 2019
CKD	Chronic kidney disease
DM	Diabetes mellitus
eGFR	Estimated glomerular filtration rate
ESRD	End-stage renal disease
ICU	Intensive care unit
HD	Hemodialysis
MERS-COV	Middle East respiratory syndrome coronavirus
OR	Odds ratio
PCR	Polymerase chain reaction
PRISMA	Preferred Reporting Items for Systematic Reviews and Meta-Analyses
RAAS	Renin–angiotensin–aldosterone system
RR	Relative risk
RRT	Renal replacement therapy
SARS-CoV-2	Severe acute respiratory syndrome coronavirus-2
