# Supplementary material for: COVID-19 and Acute Kidney Injury: A Systematic Review and Meta-Analysis
Source: Pathogens. 2020 Dec 15;9(12):1052. doi: 10.3390/pathogens9121052 (PMC7765425; doi:10.3390/pathogens9121052)
Supplement: Supplementary file 1 [file pathogens-09-01052-s001.pdf]

Supplementary Table S1. AKI according to severe COVID-19: Comorbidities.

Authors	Diabetes Mellitus, *n*	Arterial Hypertension, *n*	Cardiovascular Disease, *n*
HUANG Chaolin, *et al.*	8 (20%)	6 (15%)	6 (15%)
WANG Dawei, *et al.*	14 (10.1%)	43 (31.2%)	20 (14.5%)
XU Yonghao, *et al.*	13 (28.9%)	21 (46.7%)	6 (13.3%)
WAN Suxin, *et al.*	12 (8.9%)	13 (9.6%)	7 (5.2%)
LI Qiang, *et al.*	30 (9.2%)	78 (24%)	18 (5.5%)
XU Shen, *et al.*	147 (41.4%)	125 (35.2%)	NA
LI Zhen, *et al.*	NA	NA	NA
ZHAO Wen, *et al.*	6 (7.8%)	16 (20.8%)	9 (11.7%)
WANG Luwen, *et al.*	18 (15.5%)	43 (37.1%)	NA
ZHANG Guqin, *et al.*	22 (10%)	54 (24.4%)	22 (10%)
LI Xiaochen, *et al.*	83 (15.1%)	166 (30.3%)	34 (6.2%)
JIANG Xiufeng, *et al.*	9 (16.4%)	17 (30.9%)	2 (3.6%)
HONG Kyung, *et al.*	9 (9.2%)	30 (30.6%)	11 (11.2%)
PEI Guangchang, *et al.*	76 (22.9%)	107 (32.2%)	NA
GUAN Wej, *et al.*	81 (7.4%)	165 (15%)	27 (2.4%)
AGGARWAL Saurabh, *et al.*	5 (31%)	9 (57%)	7 (44%)
HU Ling, *et al.*	47 (14.6%)	105 (32.5%)	41 (12.7%)
CHAN Lili, *et al.*	800 (24.7%)	1,193 (36.9%)	281 (8.7%)
ZHENG Yi, *et al.*	8 (23.5%)	22 (64.7%)	4 (11.8%)
YANG Luhuan, *et al.*	21 (10.5%)	45 (22.5%)	11 (5.5%)
ARGENZIANO Michael, *et al.*	333 (39.1%)	525 (61.7%)	206 (24.2%)
SULEYMAN Geehan, *et al.*	156 (43.9%)	258 (72.7%)	102 (28.7%)

Supplementary Table S2. AKI and death risk in COVID-19: Comorbidities.

Authors	Diabetes Mellitus, *n*	Arterial Hypertension, *n*	Cardiovascular Disease, *n*
YANG Xiaobo, *et al.*	9 (17%)	NA	5 (10%)
LU Zhibing, *et al.*	14 (11.4%)	41 (33.3%)	20 (16%)
ZHOU Fei, *et al.*	36(19%)	58 (30.4%)	15 (8%)
DENG Yan, *et al.*	26 (11.5%)	58 (25.8%)	17 (7.5%)
CHENG Yichun, *et al.*	100 (14.3%)	233 (33.3%)	NA
LUO Xiaomin, *et al.*	57 (14.1%)	113 (28%)	36 (8.9%)
CAO Wen, *et al.*	NA	NA	20 (32.7%)
XU Shen, *et al.*	147 (41.4%)	125 (35.2%)	NA
CHEN Tao, *et al.*	47 (17.1%)	93 (33.9%)	24 (8.8%)
WANG Lang, *et al.*	54 (15.9%)	138 (40.7%)	53 (15.7%)
RICHARDSON Safiya, *et al.*	1808 (33.8%)	3026 (56.6%)	966 (16.9%)
PEI Guangchang, *et al.*	76 (22.9%)	107 (32.2%)	NA
CHAN Lili, *et al.*	800 (24.7%	1193 (36.9%)	281 (8.7%)
WANG Dawei, *et al.*	11 (10.3%)	26 (24.3%)	13 (12.1%)
SHI Qiao, *et al.*	153 (50%)	131 (42.8%)	49 (16%)
HIRSCH Jamie, *et al.*	1797 (32.9%)	3037 (55.7%)	949 (17.5%)
YANG Kunyu, *et al.*	22 (10.7%)	67 (32.7%)	16 (7.8%)
LIM Jeong-Hoon, *et al.*	50 (31%)	77 (48%)	31 (19.4%)
ZHAO Mengmeng, *et al.*	118 (11.8%)	282 (28.2%)	60 (6%)
PELAYO Jerald, *et al.*	104 (46.6%)	180 (80.7%)	35 (15.9%)

© 2020 by the authors. Licensee MDPI, Basel, Switzerland. This article is an open access article distributed under the terms and conditions of the Creative Commons Attribution (CC BY) license (http://creativecommons.org/licenses/by/4.0/).
